# Optimization of technology steps for obtaining white cabbage DH-plants

**DOI:** 10.18699/vjgb-25-55

**Published:** 2025-07

**Authors:** A.I. Mineykina, K.S. Stebnitskaia, M.G. Fomicheva, L.L. Bondareva, A.S. Domblides, E.A. Domblides

**Affiliations:** Federal Scientific Vegetable Center, VNIISSOK, Odintsovo district, Moscow region, Russia; Federal Scientific Vegetable Center, VNIISSOK, Odintsovo district, Moscow region, Russia; Federal Scientific Vegetable Center, VNIISSOK, Odintsovo district, Moscow region, Russia; Federal Scientific Vegetable Center, VNIISSOK, Odintsovo district, Moscow region, Russia; Federal Scientific Vegetable Center, VNIISSOK, Odintsovo district, Moscow region, Russia; Federal Scientific Vegetable Center, VNIISSOK, Odintsovo district, Moscow region, Russia

**Keywords:** white cabbage, Brassica oleracea L., DH-plants, in vitro microspore culture, androgenesis, doubled haploids, acidity of nutrient medium, shaker platform, flow cytometry, SSR-analysis, капуста белокочанная, Brassica oleracea L., DH-растения, культура микроспор in vitro, андрогенез, удвоенные гаплоиды, кислотность питательной среды, платформа-шейкер, метод проточной цитометрии клеточных ядер, SSR-анализ

## Abstract

White cabbage is one of the economically important crops among the representatives of the genus Brassica L. To create highly productive F1 hybrids with improved characteristics, the breeders need genetically diverse breeding material, which takes a long time to produce. It is possible to significantly accelerate this stage of breeding by obtaining doubled haploids (DH-plants). The lack of standardized, efficient and reproducible protocols for in vitro cultivation of different plant species, covering several factors and their interactions, often hinders the practical implementation of the method. Plant material, cultivation conditions and composition of nutrient media are determinants of embryogenesis efficiency. As a result of this study, the protocol for obtaining doubled haploids in in vitro culture of isolated microspores was optimized for late maturing white cabbage. The optimal bud size for introduction into in vitro culture varied from 3.5 to 5.0 mm. For the studied genotypes, the combined effect of high-temperature stress at 32 °C for 48 h and pH 5.8 stimulated the highest embryoid yield. The use of 3.5 g/L phytogel as a gelling agent was not effective. The use of flow cytometry allowed for separation of doubled haploids (69.8 %) from haploids (8.4 %), triploids (1.5 %) and tetraploids (20.3 %) at an early stage of development. Molecular genetic analysis with polymorphic microsatellite loci (SSR-analysis) confirmed the haploid origin of the diploid regenerant plants.

## Abstract (ru)

Одна из экономически важных сельскохозяйственных культур среди представителей рода Brassica L. – капуста белокочанная. Для создания высокопродуктивных F1 гибридов с улучшенными показателями необходима генетически разнообразная база выровненного материала, получение которого занимает продолжительную часть селекционного процесса. Ускорить этот этап селекции можно за счет получения удвоенных гаплоидов (DH-растений). Отсутствие стандартизированных, эффективных и воспроизводимых протоколов для культивирования in vitro разных видов растений, охватывающих несколько факторов и их взаимодействие, часто препятствует практической реализации метода. Растительный материал, условия культивирования и состав питательных сред являются определяющими факторами эффективности эмбриогенеза. В результате проведенного исследования оптимизирован протокол получения удвоенных гаплоидов в культуре изолированных микроспор in vitro для капусты белокочанной позднеспелого срока созревания. Оптимальный размер бутонов позднеспелой капусты белокочанной для введения в культуру in vitro варьировал от 3.5 до 5.0 мм. Для изученных генотипов капусты белокочанной совместное влияние высокотемпературного стресса при 32 °С в течение 48 ч и рН питательной среды 5.8 способствовало наибольшему выходу эмбриоидов. За счет использования платформы-шейкера при режиме 40 об./мин достигнуто ускорение развития эмбриоидов до семядольной стадии на 7–10 суток. Благодаря подобранному комплексу условий для успешного эмбриогенеза из микроспор капусты белокочанной достигнут выход эмбриоидов до 273.6 ± 32.2 шт./чашку Петри. Применение проточной цитометрии позволило отделить удвоенные гаплоиды (69.8 %) от гаплоидов (8.4 %), триплоидов (1.5 %) и тетраплоидов (20.3 %) на ранней стадии развития. Молекулярно-генетический анализ с использованием микросателлитных маркеров (SSR-анализ) подтвердил гаплоидное происхождение диплоидных растений-регенерантов.

## Introduction

Among the variety of vegetable crops belonging to the genus Brassica L., the most popular among consumers is white cabbage (Brassica oleracea L. var. capitata). In the Russian Federation this crop accounts for 14.3 % of the area occupied by vegetable crops in the open field. In the recent years, import substitution is a pressing issue in the Russian Federation. The Doctrine of Food Security of the Russian Federation (the document was approved by Presidential Decree No. 20 of January 21, 2020) strategic plan includes the task of expanding export potential in the vegetable growing industry. In this regard, vegetable producers have a great need for F1 hybrids, as they are economically advantageous for cultivation in terms of quality and resistance to adverse environmental factors.

Genetic homogeneity of parental lines is required for the development of hybrids. It can be achieved by inbreeding over several generations. Since the white cabbage is a crosspollinated crop with a two-year development cycle, it takes 12–14 years to obtain a homogenous line by conventional breeding.

In vitro culture of isolated microspores is one of the advanced biotechnological tools for obtaining homozygous lines – doubled haploids. This technology enables to achieve homozygosity in one generation. Due to genetic homogeneity, doubled haploids can be used not only in practical breeding, but also for basic research, for instance, for genetic transformation and induced mutagenesis.

The first successful experiments on the cabbage microspore culture were carried out in the early 1980s (Lichter, 1982). Then, a basic protocol for rape microspore culture was developed, which serves as a basis of DH technology for Brassica plants (Pechan, Keller, 1988). At present, this technology is being actively developed, but a high level of efficiency is required for its full-fledged inclusion in the breeding process. Plant genotype, cultivation conditions and nutrient medium ingredients are determinants of the quality and quantity of plant material obtained in any in vitro cell culture protocol. Many strategies have been implemented worldwide to improve cell culture protocols for plants of the Brassicaceae family. Important advances have been observed in all major members of the genus Brassica. Due to the high responsiveness of some species, the main factors that have impact on the induction of embryogenesis in B. napus (Weber et al., 2005), B. rapa (Ferrie et al., 1995; Gu et al., 2003; Shumilina et al., 2020), B. carinata (Barro et al., 2003), B. juncea (Prem et al., 2008) have been studied. For white cabbage, such studies are sparse due to lower responsiveness in different genotypes and low embryo yield (Cao et al., 1990; Rudolf et al., 1999; Bhatia et al., 2018).

It is known that the in vitro culture of isolated microspores is based on the ability of microspores to switch from the gametophytic to the sporophytic developmental pathway under the influence of certain factors. For plants of the genus Brassica, this ability is observed in microspores at the late one-cell stage and in pollen grains at the early two-cell stage (Pechan, Keller, 1988; Kott, 1998). In more recent studies, the viability of microspores and the embryoid yield have been shown to be dependent on the bud size, which helps to reduce the selection time when a large amount of plant material is being processed (Takahata, Keller, 1991; Bhatia et al., 2018; Kozar et al., 2022). The proper selection of the bud size ensures the homogeneity of the microspore population in terms of developmental stage, which is key to the success of this technology (Cristea et al., 2020).

Other key factors affecting the induction of embryogenesis are bud cold pretreatment and microspore heat shock during the first days of cultivation (Takahata, Keller, 1991; Bhatia et al., 2018). In all tested protocols, temperature shock initiated microspore division. According to studies (Pechan, Smykal, 2001), temperature treatment at 32 °C for 1–4 days is a required condition for microspore induction in B. napus. Thus, the combination of cold pretreatment (4 °C) for 1 or 2 days and heat shock (32.5 °C) for 1 day significantly enhanced microspore embryogenesis in broccoli (Yuan et al., 2012), and 32.5 °C for 1 or 2 days was optimal for white cabbage (Yang et al., 2013).

The liquid nutrient NLN medium with 13 % sucrose was developed in 1982 (Lichter, 1982) and has been used in microspore cultivation protocols for multiple cultures. Studies have shown that the acidity of the medium has a significant influence on embryogenesis. pH varies between 5.6 and 6.6 for different genotypes of cabbage cultures (Yuan et al., 2012). To increase the viability of induced microspores and developing embryoids, it is also beneficial to add activated charcoal to the medium (Prem et al., 2008).

An unambiguously positive response was observed when a shaker platform at 40 to 50 revolutions per minute was used for culturing microspores on the liquid medium. This increased the formation of Brassica rapa L. ssp. chinensis embryoids by 11.6–69.37 %, as well as shortened the culturing time by 1–4 days and accelerated plant regeneration (Yang et al., 2013). It is also important to support further embryoid and plantlet development. MS solid medium (Murashige, Skoog, 1962) is most commonly used for propagation, while S. Yuan and colleagues (Yuan et al., 2012) use 1/2 MS medium with 50 % salt content for rooting (Yang et al., 2013).

The procedure for obtaining DH lines includes two main steps: induction of embryogenesis and chromosome doubling. The second step is necessary for the practical application of the obtained regenerants, since plants are sterile before genome doubling. At present, the mechanism underlying spontaneous chromosome doubling is unclear in many cases, and its efficiency varies greatly among species and cultivars of the same species (Kasha, 2005). A study by J.C. da Silva Dias and coauthors found 43–88 % spontaneous diploidization in broccoli and 7–91 % spontaneous diploidization in other cabbage species (da Silva Dias, 2003). Since plants with different ploidy can occur among regenerants, it is necessary to analyze the ploidy level of all obtained plants.

Early studies have shown that embryogenic ability in cabbage crops is generally a quantitatively inherited trait controlled by several genes that varies among cultivars and genetic groups (Zhang et al., 2003; Kitashiba et al., 2016; Ji et al., 2023). Thus, the selected optimal factors for successful in vitro cultivation of microspores are suitable for a particular genotype. However, there is no universal cultivation protocol. Identification and modification of potentially interacting factors would help to improve embryoid yield, which is particularly important for low-responsive genotypes.

The aim of this study is to improve the basic protocol of isolated microspore culture for late-maturing genotypes of white cabbage by identifying optimal cultivation conditions.

## Materials and methods

Material and growing conditions. Eight varieties of latematuring white cabbage (Brassica oleracea var. capitata L.) from the collection of the LLC “Agrofirma Poisk” (No. 2403, 2404, 2405, 2406, 2407) and FSBSI FSVC (No. 127, 303, 360) were used. All genotypes had a maturity period of 160 to 180 days from sprouting and represented valuable breeding samples of different genetic origin, selected for economically valuable traits.

Donor plants were grown in a climatic chamber at 19 °C, illumination of 65 μmol · m–2 · s–1 and photoperiod of 16 h – day, 8 h – night. Plant vernalization was carried out at 6 °C in the dark for three months. At the end of vernalization, at the acclimatization stage, the growing vessels with plants were placed in a climatic chamber to obtain inflorescences. During 15–35 days, the temperature was gradually increased from +8 °C to 16±2 °C under the regime of 16 h – day, 8 h – night and illumination 65 μmol · m–2 · s–1.

Culture of isolated microspores in vitro. At the first stage, buds containing the maximum number of microspores at the stages potentially capable of embryogenic development from late uninucleate to early binucleate were selected by linear size. Stages were identified by staining anthers with a differential dye (Alexander, 1969) and observing them under an Axio Imager A2 microscope (Zeiss, Germany). A microspore suspension was prepared from the selected buds so that microspores from 1 bud were placed in 1 ml of NLN medium (Lichter, 1982) with 13 % sucrose and pH 5.8; 6.0; 6.1; 6.2; 6.4, depending on the experiment. For this purpose, buds were collected at the beginning of donor plant flowering and sterilized for 30 s in 96 % ethanol. Then, they were sterilized for 15 min in a sodium hypochlorite commercial solution (“Belizna”, Russia) diluted by sterile distilled water in a 1:1 ratio with the addition of Tween 20 (Panreac, Spain) (1 drop per 100 ml of solution), followed by three 7 min washes in sterile distilled water.

Sterile buds were placed in the glass flasks with the NLN medium and magnets on a magnetic stirrer (BioSan, Latvia). The resulting microspore suspension was passed through a 40 μm nylon filter and then centrifuged at 920g for 5 min using an Eppendorf 5804R centrifuge (Germany). Microspores were washed twice. After isolation and washing, microspores were placed in 60 mm Petri dishes. The sterile solution of activated charcoal and agarose (Sigma-Aldrich, USA) (1 g of activated charcoal per 100 ml of 0.5 % agarose solution) was melted in a microwave oven. Then 3–4 drops of this solution were added to each Petri dish. Petri dishes were then incubated in the dark at 32 °C shock temperature for 1, 2, 3 days, followed by 25 °C incubation in the dark at 40 rpm in a shaker incubator (New Brunswick Innova® 44/44R Eppendorf, Germany) until embryoids reached the cotyledonary stage. All experiments were performed in triplicate.

Plant regeneration. Embryoids that reached the cotyledonary stage were transferred to the solid MS medium with 2 % sucrose and 0.7 % agar, pH 5.8, supplemented with 1 mg/L 6-benzylaminopurine (6-BAP), 0.1 mg/L 1-naphthylacetic acid (NAA), or 0.1 mg/L gibberellic acid (GA). Shoots were subcultured every 4 weeks on the same medium without the addition of growth regulators. The cultivation was performed on racks under mixed illumination of two types of fluorescent lamps: OSRAM Fluora L36W/77 (predominantly blue and red spectrum) and Philips 36W/54-765 (predominantly white spectrum), at a total illumination of 24 μmol · m–2 · s–1 at 16 h day/8 h night and 24 ± 2 °C.

Adaptation of plants to in vivo conditions. The regenerant plants with developed leaves, stems and roots were transplanted into 8-cm-diameter pots with peat and perlite (7:3) for adaptation to in vivo conditions. To improve adaptation and maintain high humidity during the first week after transplanting into the soil, the regenerant plants were covered with perforated plastic transparent cups tightly adhering to the substrate. Then the cups were gradually lifted and removed. Adaptation took place in a climate room with the same parameters as for donor plants.

DNA extraction for PCR analysis from regenerant plants of white cabbage. Young leaves of each plant were ground in 200 μL of CTAB buffer using tungsten carbide beads (3 mm in diameter) and a TissueLyser II homogenizer (Qiagen, Germany) (1560 oscillations/min, duration 1.7 min) to a suspension. After grinding, 15 μL of proteinase K was added to each sample. Further DNA extraction was performed by CTAB method using Sorb-GMO-B reagent kit (Syntol, Russia) according to the manufacturer’s protocol. The final purity and concentration of total DNA were determined using a spectrophotometer (Smart Spec Plus, Bio-Rad, USA). The obtained ratio OD260/280 = 1.6–1.8 corresponded to the pure DNA solution. Preparations of isolated DNA were stored in a freezer at –70 °C.

PCR analysis of white cabbage regenerant plants. Nine microsatellite loci (Table 1) with known primer sequences for amplification showing a primer PIC of at least 0.5 (Tonguç, Griffiths, 2004; Louarn et al., 2007) were used for microsatellite analysis.

**Table 1.**
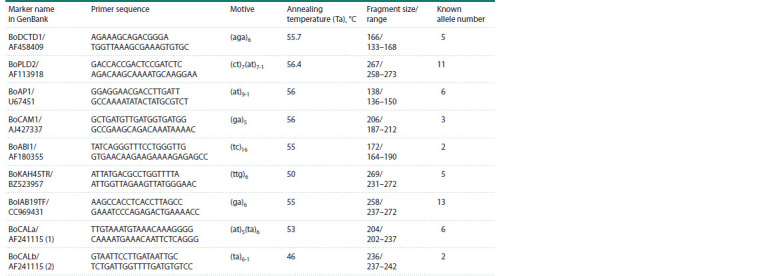
Characteristics of microsatellite loci

Basic PCR was performed in a volume of 25 μL, including 1x PCR buffer B, 2.5 mM MgCl2, 0.25 mM of each dNTP, 0.3 μM of each primer, 1.5 units of Taq DNA polymerase (Syntol, Russia) and 3 μL of DNA from each plant sample tested. The C1000 Touch instrument (BioRad, USA) was used for amplification. The basic amplification protocol consisted of a denaturation step of 2–5 min at 92–95 °C; an annealing step of 30 sec at temperatures from 52 to 58 °C; and an elongation step of 30 sec to 1 min at 72 °C. The program was designed for 35 cycles of amplification.

PCR products were separated by vertical electrophoresis using the Mini-PROTEAN Tetra Cell system (BioRad, USA) in a 6 % polyacrylamide gel. After electrophoresis, gels were stained with SYBR™ Safe DNA Gel Stain (Invitrogen, USA) according to the manufacturer’s instructions and documented using the ChemiDoc XRS+ system (BioRad, USA).

The sizes of the amplified fragments were determined by comparison with Thermo Scientific GeneRuler 100 bp Plus DNA Ladder molecular mass marker (Thermo Fisher Scientific Baltics UAB, Lithuania). The obtained digital photographs of amplification products were analyzed using ImageLab 3.0 software (BioRad, USA).

Ploidy determination by flow cytometry. Young healthy leaves were chopped with a razor blade in 300 μL of Galbraith buffer (45 mM MgCl2, 20 mM MOPS, 30 mM sodium citrate, 0.1 % Triton X-100, pH 7.0) on ice supplemented with 50 μg/ mL RNase I (Syntol, Russia). The sample was then filtered through a 30 μm nylon filter. Then, propidium iodide (Sigma, USA) was added to a final concentration of 50 μg/mL. DNA content was determined by the fluorescence intensity of propidium iodide staining on a Beckman Coulter CytoFLEX flow cytometer with the B2-RO-V2 kit (Beckman Coulter, USA) with a 532 nm laser light source.

Histogram visualization and data processing were performed using CytExpert 2.4 software (Beckman Coulter, USA).

Donor plant diploid samples were used as an external standard to determine ploidy and DNA content. The ploidy was determined by the index of the difference between the diploid standard and the sample peaks:

Index = the average peak of the sample the average peak of the standard .

DNA content (2C, pg) was calculated according to the formula:

2С = the average peak of the sample the average peak of the standard × 2С of standard

Statistical analysis. Statistical analysis was performed using analysis of variance (one-way, two-way ANOVA) and mean values were compared using Duncan’s multiple range test (DMRT) with 95 % probability. Statistical analysis was performed using Statistica 8.0 (Statsoft, www.statsoft.com).

## Results

Stages of white cabbage embryogenesis

**Fig. 1.**
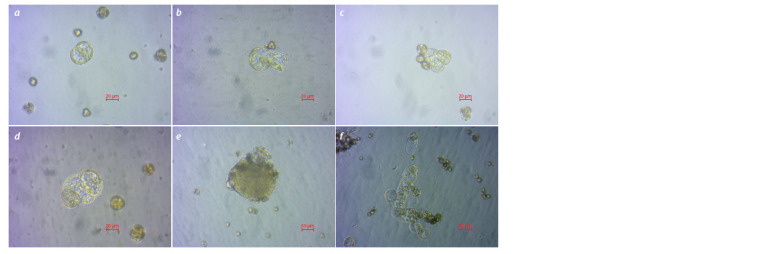
Microspore division and embryoid formation in white cabbage. а – the first microspore division (day 1); b – day 2; c – day 3; d – day 6; e – globule formation (14 days); f – a suspensor-like structure.

**Fig. 2.**
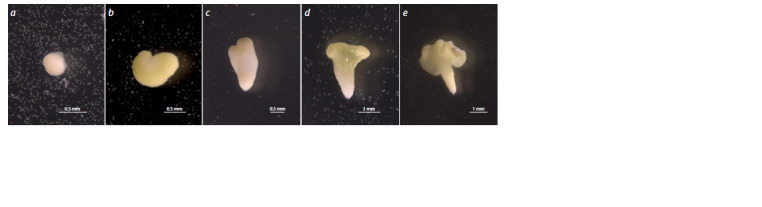
Growth and development of white cabbage embryoids in the microspore culture in vitro а – a globular stage after 16 days of cultivation; b – heart-shaped stage after 20 days of cultivation; c – heart-shaped stage on day 25; d – an embryoid at the cotyledonary stage after 30 days; e – an embryoid with expanded cotyledons after 30 days of cultivation.

After isolation, white cabbage microspores were subjected to high-temperature stress at 32 °C for 1–2 days, after which microspore division was observed (Fig. 1a, b). For all studied samples of late-maturing white cabbage, multicellular structures could be observed on day 15 of cultivation. Their further development could occur by different embryoid formation pathways. The most common is the formation of globules by dividing cells. Further, similar to zygotic embryoids in vivo, during the transition to the heart-shaped stage, radial symmetry changes to bipolar symmetry with the development of two future cotyledons (Fig. 2). Another way of embryoid formation is through suspensor-like structures. In such structures, we observed transverse division of daughter cells, resulting in the formation of a suspensor shaped as a long filament (Fig. 1f ). The embryoid formation pathways have been investigated in more detail in other Brassicaceae crops in different studies (Tang et al., 2013; Kozar et al., 2021).

**Fig. 3.**
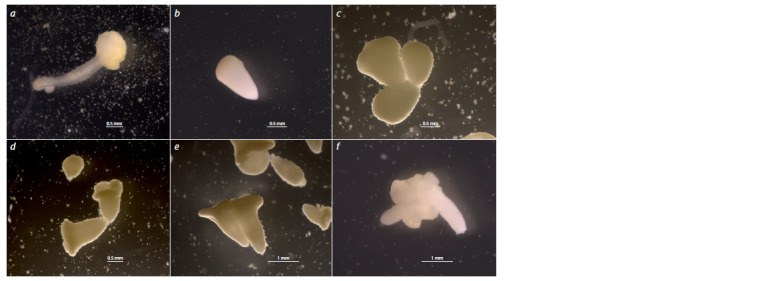
Abnormal development of white cabbage embryoids in in vitro microspore culture. а – an embryoid with no pronounced hypocotyl; b – an embryoid without formed cotyledons; c–f – conjoined twin embryoids.

Along with normal embryoids, in all genotypes we also observed abnormally developed embryoids characterized by the absence of cotyledons and hypocotyl (Fig. 3a, b), as well as various twin forms (Fig. 3c–f ). The percentage of abnormal embryoids ranged from 1 % to 15 % depending on the number of embryoids formed per Petri dish (embryoid yield). The percentage of abnormal embryoids increased in highly responsive genotypes.

The bud size affects embryogenesis efficiency

The predominant stage of microspore development in culture, which correlates with the bud size, was a determinant of embryogenesis efficiency in white cabbage. It was noted that it is impossible to determine a single optimal bud size for all genotypes. Before introduction into in vitro culture, it is necessary to determine for each genotype, which bud linear size has the maximum percentage of microspores at the optimal developmental stages for androgenesis induction, since genotypes have different phenotypic features, including bud form. A round or elongated form of the bud will have a significant effect on the range of bud lengths suitable for microspores isolation.

In preliminary experiments, it was determined that embryoid induction per Petri dish was significantly reduced or inhibited, if the bud length range exceeded 1 mm in a bud sample. This was due to the fact that many microspores/pollen grains were introduced into the in vitro culture, which died by day 10–14 of cultivation and had a toxic effect on the culture. The male gametophyte stages optimal for embryogenesis were in bud samples varying by 0.5 mm in length.

In eight genotypes, we isolated several bud groups differing in length and studied the embryoid yield (Table 2). For all genotypes, the maximum embryoid yield was obtained only in one of the groups, which confirms the need to select buds by size with a variation of no more than 0.5 mm in the sample. Only for genotype No. 2404 the optimum bud size was 3.5–3.9 mm. For two genotypes (No. 2403 and 303), the optimum size was 3.9–4.5 mm, and for five genotypes, (No. 2405, 2406, 2407, 127, 360) it was in the range of 4.5–5.0 mm. The highest embryoid yield was achieved for genotype No. 2406 from the 4.5–5.0 mm buds and averaged 273.56 ± 32.21 embryos/Petri dish (Table 2).

**Table 2.**
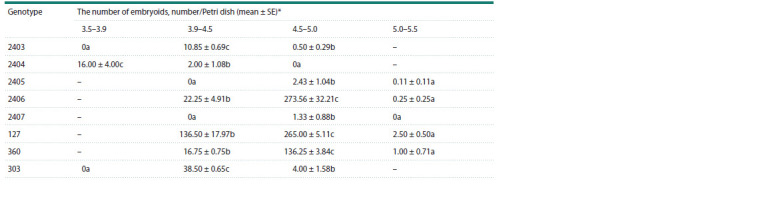
White cabbage embryoids yield in in vitro microspore culture depending on bud size Notе. “–” – microspores at the optimal developmental stage were absent in the study variants. * Here and in the Table 3: values in a row within a genotype with the same lowercase letter (a–c) are not significantly different with 95 % probability, according to Duncan’s multiple range test.

The effect of temperature treatment, pH of the medium and their combined effect on microspores embryogenesis

**Fig. 4.**
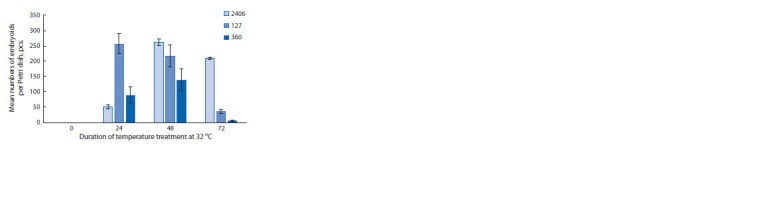
The effect of the shock temperature treatment duration on embryoids yield in the highly responsive white cabbage genotypes No. 2406, 127, and 360 (from left to right: constant 25 °C, 24 h – 32 °C, 48 h – 32 °C, 72 h – 32 °C).

No embryoids were formed when microspores were cultured at a constant temperature of 25 °C in all genotypes used in the study. Short-term 32 °C shock temperature treatment proved to be a key factor in reprogramming microspores to the sporophytic path of development with the formation of embryoids for all studied white cabbage genotypes. The duration of high temperature treatment had a significant effect on embryoids yield. The maximum embryoid yield for genotype No. 2406 was achieved after 48 h treatment. One-day high temperature treatment also initiated embryogenesis, but it was insufficient for the majority of potentially embryogenic microspores in the studied genotype (Supplementary Materials, Fig. S1)1. For genotypes No. 127 and 360, the difference between embryoid yield under high-temperature treatment for 24 and 48 h was insignificant. When the treatment time was increased up to three days, there was a significant decrease in embryoids yield for all studied white cabbage genotypes (Fig. 4). The genotypes characterized by low responsiveness had no embryoids in this variant of the experiment (Table S2).


Supplementary Materials are available in the online version of the paper: https://vavilov.elpub.ru/jour/manager/files/Suppl_Minej_Engl_29_4.pdf


The use of the medium with different pH on four white cabbage genotypes showed a significant effect of medium acidity on embryoid yield. At the same time, all genotypes responded differently to the different pH (Table 3). In our experiments, we used nutrient media with the most common pH values for in vitro cultivation (Yuan, 2012) (5.8; 6.1; 6.4). Within each genotype, we observed a significant shift in embryoid yield relative to the pH of the medium. For genotypes No. 2403, 2405, 2407, the highest embryoid yield was achieved at pH 5.8. On the contrary, for genotype No. 2404, the medium with pH 6.1 and 6.4 increased the embryogenesis efficiency more than two times. Nutrient medium with pH 5.8 also promoted embryoid formation for this genotype but to a smaller extent.

**Table 3.**
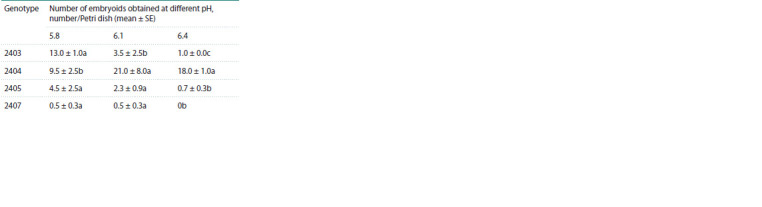
The white cabbage embryoid yield in in vitro microspore culture depending on the nutrient medium pH

For genotype No. 2403, an experiment on the combined effect of medium acidity and high-temperature treatment on embryogenesis was conducted. Two-factor analysis of variance showed that both the factor of nutrient medium acidity and the factor of high-temperature induction treatment, as well as the interaction of both factors have a significant effect on embryoids yield (Table S2). At the same time, the factor of temperature induction treatment is the main one (the influence share of 45 %). Apparently, this is the factor that triggers embryogenesis in the culture of isolated microspores. This is confirmed by the absence of embryoids in all three variants of the experiment with different nutrient medium acidity, if the microspores were not subjected to high-temperature stress and cultured at 25 °C. Temperature treatment at 32 °C promoted microspore reprogramming to the sporophytic developmental route and induced embryogenesis in all tested variants of nutrient medium acidity. At the same time, the highest embryoid yield was achieved after 48 h high-temperature treatment at pH 5.8. Increasing the duration of temperature treatment up to 2 days contributed to the increase of embryoid yield at pH 6.1, but in a smaller extent. However, at a pH value of 6.4, the duration of temperature treatment did not significantly affect embryoids yield (Table S2).

The regeneration of DH plants from embryoids

**Fig. 5.**
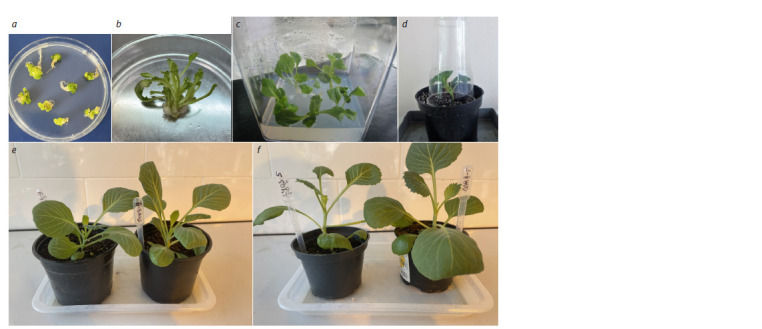
The regeneration of white cabbage No. 2403 plants from embryoids and their adaptation to ex vitro conditions. а – рembryoid outgrowth on MS medium; b – secondary embryogenesis; c – rhizogenesis; d – adaptation to ex vitro conditions; e – the diploid plants (2n) obtained from genotype No. 2403; f – the tetraploid plants (4n) obtained from genotype No. 2403.

Embryoids with cotyledons were first transplanted from liquid medium to solid MS (Fig. 5a), supplemented with 20 g/L sucrose, 7 g/L agar, BAP (1 mg/L), NAA (0.1 mg/L), and GA (0.1 mg/L) to induce shoot formation (Fig. 5b). One month later, the developing adventitious shoots were transplanted for rooting onto the solid hormone-free MS medium containing 20 g/L sucrose and 7 g/L agar or 3.5 g/L phytogel. When planted on the medium with phytogel, callus outgrowth at the base of the shoot with no root formation was observed. On nutrient media with agar, shoots quickly grew a well-developed root system (within 7–10 days) (Fig. 5c).

Identification of regenerant plant ploidy

The regenerant plant ploidy was determined at the seedling stage (5–8 leaves) by flow cytometry of isolated nuclei (Fig. S2). The analysis of 163 regenerant plants of the six white cabbage genotypes that successfully passed the adaptation stage showed that the average percentage of haploids was 8.4 %, doubled haploids, 69.8 %, triploids, 1.5 %, tetraploids, 20.3 %. The genotypes noticeably differed by the occurrence of different ploidy (Table 4).

**Table 4.**
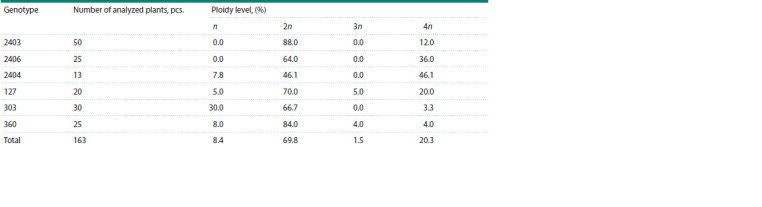
White cabbage regenerant plant ploidy

Confirmation of haploid origin of white cabbage regenerant plants

To confirm the haploid origin of diploid regenerated plants from four samples (No. 2403, 2406, 303, 360) obtained through the culture of isolated microspores, nine microsatellite loci (AJ427337, AF180355, AF241115(1), AF241115(2), AF458409, AF113918, BZ5223957, CC969431, U67451) that had been previously successfully used for genotyping of white cabbage were evaluated (Tonguç, Griffiths, 2004; Louarn et al., 2007; Domblides et al., 2020). Genetic differences between the regenerated plants and the donor genotypes were not observed in loci AJ427337, AF180355, and AF241115(1), where only one allele was obtained for all donor plant genotypes included in the study. More than four alleles were amplified with primers for the CC969431 locus, which was more than expected. Therefore, this marker was not taken for further difference assessment of the studied samples. Amplification with loci BZ5223957 and AF241115(2) revealed a polymorphism between donor plants and obtained regenerant plants only in genotypes No. 2406 and 303.

**Fig. 6.**
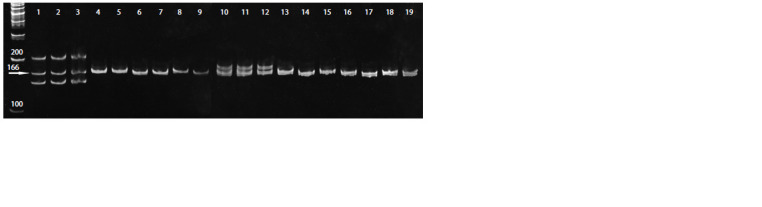
The electrophoregram of DNA amplification results of white cabbage plants with microsatellite locus AF458409. Here and in the Figure 7: the numbers: 1–3 – donor plants of genotype No. 2406; 4–9 – diploid regenerant plants obtained in the isolated microspore culture of No. 2406; 10–12 – donor plants of genotype No. 2403; 13–19 – diploid plants – regenerants obtained in the isolated microspore culture of No. 2403.

A polymorphism in all four genotypes was identified in loci AF458409, AF113918 and U67451. For example, amplification of the AF458409 locus revealed three alleles for No. 2403 and two alleles for No. 2406 in donor plants, whereas only one allele was observed in diploid regenerated plants (Fig. 6).

**Fig. 7.**
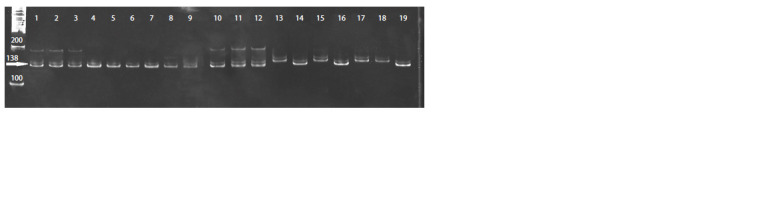
The electrophoregram of DNA amplification results of white cabbage plants by microsatellite locus U67451.

The U67451 locus showed two alleles in donor plants No. 2406, 303 and 360 and three alleles in donor plants No. 2403, while only one allele was present in regenerant plants (Fig. 7).

The absence of identity in SSR profiles of donor plants and regenerated plants was also observed for the microsatellite locus AF113918, where regenerated plants had a reduced number of alleles in SSR profiles compared to their donor plants.

Thus, genetic analysis with use of microsatellite markers enables to confirm the haploid origin of regenerated plants from microspores. When analyzing the profiles of three microsatellite loci AF458409, AF113918 and U67451, the absence of similarity in SSR markers between donor plants and regenerated plants was shown, and the reduced number of alleles in DH plants of white cabbage was also observed. In the case of implementation of in vitro culture of isolated anthers to obtain DH plants, this stage of the technology should be required to have 100 % accuracy, so that it is possible to separate true doubled haploids from diploid regenerated plants, which may have originated from anther somatic tissues.

## Discussion

Over the last decades, significant progress has been made in the development of cell technologies for Brassica plants. They are based on the optimization of cultivation conditions and the application of manipulations that increase the embryoid yield.

The selection of buds at the optimal stage of microspore development determines the success of embryogenesis in any in vitro cultivation protocol. According to literature data, microspores at the late uninucleate and early bicellular stages have the highest probability of embryoid formation in vitro (Kott et al., 1988; Pechan, Keller, 1988). The stage of microspore development correlates with bud size, which allows us to select material based on this characteristic (Fan et al., 1988; Huang et al., 1990). Studies for different members of the Brassicaceae family have shown that the optimal bud size differs between species, different genotypes within one species and individual plants within one genotype. The optimal bud sizes of the studied genotypes were established experimentally – 4–5 mm for broccoli (Takahata, Keller, 1991), 4–6 mm for cauliflower (Gu et al., 2014), 4.1–5.0 mm for red cabbage (Mineykina et al., 2021), 3.0–3.1 mm for sarepta mustard (Ali et al., 2008), 2.5–3.5 mm for white cabbage (Yuan et al., 2012; Tuncer et al., 2016), 4.5–4.6 mm for white cabbage of Indonesian origin (Winarto, da Silva, 2011).

In early studies, it was shown that a high percentage of nonembryogenic microspores in the culture resulted in increased levels of autotoxins in the medium affecting embryoids development. The negative effect of the toxins was correlated with the presence of bicellular microspores in the culture (Kott et al., 1988). In more recent studies (Duijs et al., 1992), good results were obtained in variants with 10 to 40 % of bicellular pollen in buds. We observed that the microspore developmental stage is a limiting factor in in vitro culture of white cabbage. Since cabbage exhibits pronounced asynchronous development of microspores within the bud, their selection with a certain limit of size variation maximizes the coverage of potentially embryogenic microspores

An early study (Lighter, 1989) modified the microspore culturing protocol for the Brassicaceae family. The addition of activated charcoal to the cultured medium and the use of a shaker in microspore culture helped to minimize factors affecting cell destruction and suppression of cell division. Studies J.C. da Silva Dias, (1999) also indicate an increase in the embryogenesis of different cabbage genotypes when activated charcoal is used in the culture medium. A reduction in cultivation time by 1–4 days using a shaker platform was observed when Chinese cabbage microspores were cultured in liquid medium (Yuan et al., 2012).

In our studies, to increase the survival rate of white cabbage microspores and inhibit oxidation products that negatively affect cell division, we used activated charcoal in all variants as an integral element of the protocol. The use of a shaker platform allowed us to accelerate the embryoid development, thereby increasing the efficiency of the protocol by reducing the culture time in the liquid nutrient medium. As a result, more embryoids reached the cotyledonary stage 7–10 days faster, which is especially important for highly responsive genotypes that have a non-uniform embryoid maturation.

Stress is the most important condition during the transition of microspores from the gametophytic to the sporophytic pathway (Touraev et al., 1996). Stress can be applied both in vivo and in vitro. The most common type of stress for cabbage crops is exposure of buds and inflorescences to low positive temperatures (Gu et al., 2014) and short-term microspore heat shock in the in vitro culture (Custers et al., 1994).

Pretreatment with low positive temperatures does not promote reprogramming of microspore development but effectively maintains microspore viability (Żur et al., 2009). Heat treatment of isolated microspores in a range from 30 to 40 °C with time exposure from 1 to 3 days is most commonly used as an inducing factor of embryogenesis (Takahata, Keller, 1991; Duijs et al., 1992; Ferrie, Caswell, 2011). Some authors have noted that the duration of temperature treatment affects the number of developing embryoids (Telmer et al., 1992; Custers et al., 1994; Cordewener et al., 1995; Simmonds, Keller, 1999). For white cabbage of Indonesian origin (Winarto, da Silva, 2011), exposure to 30.5 °C for 48 h followed by continuous cultivation at 25 °C was found to be a successful approach. However, 30 % of embryoids in that study had abnormal cotyledon and no hypocotyls. In a study (Tuncer et al., 2016), the authors studied the effect of temperature shock on the induction of embryogenesis and embryoid development in B. oleraceae of Turkish origin. The authors observed a positive effect of 32 and 35 °C 2-day treatment on the induction of embryogenesis. However, the embryoid development was impeded in this study, and white cabbage plants did not regenerate.

To understand the molecular regulation of embryogenesis induced by high-temperature treatment, H. Su et al. (2019) performed a proteomic study. They found that the 32 °C hightemperature shock for 24 h induced changes in the expression of specific proteins in an in vitro culture of isolated cabbage microspores.

In addition to the fact that high-temperature stress is an effective trigger for switching microspore development to the sporophytic pathway, it also has a negative effect on cell division. Studies A. Zeng et al. (2015) showed massive white cabbage microspore death (80–90 %) after 3 days of in vitro cultivation. The authors observed microspore death after 24 h of shock temperature treatment at 32.5 °C, presumably caused by increased levels of reactive oxygen species (ROS) and the associated oxidative stress affecting cell viability and metabolism. According to the results of I. Żur et al. (2009), the lethal effect of high temperature during the induction of triticale microspore embryogenesis is associated with a sharp decrease in the enzymatic activity of all studied antioxidants. The authors suggest that high temperature stress induced oxidative stress, and cells in a nitrogen-carbohydrate starvation environment (Kyo, Harada, 1986) were unable to activate defense responses. The reduction of ROS levels was promoted by the use of ascorbic acid as an antioxidant in microspore culture (Zeng et al., 2015). However, the use of ascorbic acid in in vitro culture does not always have a positive effect and depends on its concentration (Rodriguez-Serrano et al., 2012; Hoseini et al., 2014).

In a study I. Barinova et al. (2004), the effects of carbohydrate stress induced by high pH values of the medium were observed in the cultivation of tobacco microspores. Analysis of sucrose metabolism at different pH showed that the activity of invertase (EC 3.2.1.26) in microspores was the highest at pH 5.0 and strongly decreased at higher pH, resulting in slower sucrose cleavage. These data suggested that isolated microspores cannot metabolize carbohydrates at higher pH and undergo starvation stress, which in turn triggers sporophytic development.

Our studies confirm the inducing effect of high-temperature stress at 32 °C on the white cabbage microspore development via the sporophytic pathway. The effect of the nutrient medium acidity on the embryoid yield depends on the duration of microspore high-temperature treatment.

Among the techniques used to obtain doubled haploids, only isolated microspore culture can ensure that the embryoids develop from haploid cells, whereas anther and unfertilized ovule cultures can include somatic tissues. The process of callus and embryoid formation as well as subsequent plant regeneration from somatic diploid tissues of anther walls is a well-known fact. Molecular analysis has been successfully used to distinguish true doubled haploid lines originating from gametes from regenerant plants of somatic origin. The use of molecular markers to screen pepper plants obtained via in vitro culture is known from the literature (Gyulai et al., 2000). A. Cousin and M.N. Nelson (2009) used eight microsatellite markers to confirm homozygosity and the haploid cell origin of rape regenerant plants obtained in microspore culture.

SSR analysis has also been successfully used to assess heterozygosity and determine the origin (gametophytic or somatic) in lemon (Yahyaoui, Germanà, 2021), cucumber (Diao et al., 2009), melon (Malik et al., 2011) and Chinese cabbage (Adamus et al., 2021). Our results also allowed us to confirm the gametophytic origin of the regenerant plants using microsatellite markers. We showed that donor plant bands are different from the regenerant plant. Moreover, the number of alleles in DH plants of white cabbage decreased compared to the control.

## Conclusion

In this study, we optimized the protocol for obtaining doubled haploids in the culture of isolated microspores in vitro for late-maturing white cabbage to achieve up to 273.6 ± 32.2 embryoids/ Petri dish. A genotype-specific multifactorial approach should be used to achieve the best results. Before introduction into in vitro culture, determination of linear bud size is required. To obtain microspores at the optimal developmental stage for embryogenesis induction, the range of bud lengths in the sample should not exceed 0.5 mm. The final yield of white cabbage embryoids is significantly influenced by the duration of temperature induction treatment and the acidity of the nutrient medium. Cultivation of the induced microspores in the dark on a shaker platform at 40 revolutions/minute allows to significantly accelerate the development of embryoids to the cotyledonary stage and to shorten this step of the protocol by 7–10 days. Flow cytometry makes it possible to determine the ploidy of the regenerant plants at an early stage of development rather quickly. The following ploidy was observed: haploids (8.4 %), doubled haploids (69.8 %), triploids (1.5 %) and tetraploids (20.3 %).

The analysis with microsatellite markers confirmed the haploid origin of diploid regenerant plants. Three microsatellite loci AF458409, AF113918, and U67451 showed that the spectra of white cabbage donor plants and regenerant plants were not identical. Moreover, a smaller number of alleles was observed in DH plants.

## Conflict of interest

The authors declare no conflict of interest.
